# Supplementary material for: Experiences of multimorbidity in urban and rural Malawi: An interview study of burdens of treatment and lack of treatment
Source: PLOS Glob Public Health. 2022 Mar 24;2(3):e0000139. doi: 10.1371/journal.pgph.0000139 (PMC10021162; doi:10.1371/journal.pgph.0000139)
Supplement: S1 Checklist — (DOCX) [file pgph.0000139.s001.docx]

**Supplementary File 1: Standards for Reporting Qualitative Research Checklist**

| No | Topic | Item | Page/line |
| --- | --- | --- | --- |
| Title and Abstract | | | |
| S1 | Title | Concise description of the nature and topic of the study Identifying the study as qualitative or indicating the approach (e.g., ethnography, grounded theory) or data collection methods (e.g., interview, focus group) is recommended | P1; lines 1-2 |
| S2 | Abstract | Summary of key elements of the study using the abstract format of the intended publication; typically includes background, purpose, methods, results, and conclusions | P2-3; lines 43-71 |
| Introduction | | | |
| S3 | Problem formulation | Description and significance of the problem/phenomenon studied; review of relevant theory and empirical work; problem statement | P4-7; lines 79-154 |
| S4 | Purpose or research question | Purpose of the study and specific objectives or questions | P7; lines 15-154 |
| Methods | | | |
| S5 | Qualitative approach and research paradigm | Qualitative approach (e.g., ethnography, grounded theory, case study, phenomenology, narrative research) and guiding theory if appropriate; identifying the research paradigm (e.g., postpositivist, constructivist/ interpretivist) is also recommended; rationale | P8; lines 158-163 |
| S6 | Researcher characteristics and reflexivity | Researchers’ characteristics that may influence the research, including personal attributes, qualifications/experience, relationship with participants, assumptions, and/or presuppositions; potential or actual interaction between researchers’ characteristics and the research questions, approach, methods, results, and/or transferability | P10-11; lines 213- 235 |
| S7 | Context | Setting/site and salient contextual factors; rationale | P8; lines 167-173 |
| S8 | Sampling strategy | How and why research participants, documents, or events were selected; criteria for deciding when no further sampling was necessary (e.g., sampling saturation); rationale | P8; lines 167-173 |
| S9 | Ethical issues pertaining to human subjects | Documentation of approval by an appropriate ethics review board and participant consent, or explanation for lack thereof; other confidentiality and data security issues | P11; lines 237-245 |
| S10 | Data collection methods | Types of data collected; details of data collection procedures including (as appropriate) start and stop dates of data collection and analysis, iterative process, triangulation of sources/methods, and modification of procedures in response to evolving study findings; | P8-9; lines 177 - 187 |
| S11 | Data collection instruments and technologies | Description of instruments (e.g., interview guides, questionnaires) and devices (e.g., audio recorders) used for data collection; if/how the instrument(s) changed over the course of the study | P8-9; lines 177 - 187 |
| S12 | Units of study | Number and relevant characteristics of participants, documents, or events included in the study; level of participation (could be reported in results) | P12; lines 246-253 |
| S13 | Data processing | Methods for processing data prior to and during analysis, including transcription, data entry, data management and security, verification of data integrity, data coding, and anonymization/de-identification of excerpts | P9; lines 181-184 and 191-198 |
| S14 | Data analysis | Process by which inferences, themes, etc., were identified and developed, including the researchers involved in data analysis; usually references a specific paradigm or approach; rationale | P9-10; lines 191-211 |
| S15 | Techniques to enhance trustworthiness | Techniques to enhance trustworthiness and credibility of data analysis (e.g., member checking, audit trail, triangulation); rationale | P10; lines 209-211 |
| Results/Findings | | | |
| S16 | Synthesis and interpretation | Main findings (e.g., interpretations, inferences, and themes); might include development of a theory or model, or integration with prior research or theory | P12-29; lines 247-612 |
| S18 | Links to empirical data | Evidence (e.g., quotes, field notes, text excerpts, photographs) to substantiate analytic findings | P12-29; lines 247-612 |
| Discussion | | | |
| S18 | Integration with prior work, implications, transferability, and contribution(s) to the field | Short summary of main findings; explanation of how findings and conclusions connect to, support, elaborate on, or challenge conclusions of earlier scholarship; discussion of scope of application/generalizability; identification of unique contribution(s) to scholarship in a discipline or field | P30-33; lines 615 -702 |
| S19 | Limitations | Trustworthiness and limitations of findings | P33; lines 695-702 |
| Other | | | |
| S20 | **Conflicts of interest** | Potential sources of influence or perceived influence on study conduct and conclusions; how these were managed | P35; 703-731 |
| S21 | **Funding** | Sources of funding and other support; role of funders in data collection, interpretation, and reporting | P35; 726-728 |
