# Supplementary material for: Experiences of multimorbidity in urban and rural Malawi: An interview study of burdens of treatment and lack of treatment
Source: PLOS Glob Public Health. 2022 Mar 24;2(3):e0000139. doi: 10.1371/journal.pgph.0000139 (PMC10021162; doi:10.1371/journal.pgph.0000139)
Supplement: S1 File — (DOCX) [file pgph.0000139.s002.docx]

**Supplementary File 1: Interview topic guide (English)**

Thank you for agreeing to participate in this interview, we are grateful for your time and appreciate your contribution to this research work. Over the course of the next hour or so, I am going to ask you a series of questions about your life and your health. There are no ‘right’ answers to these questions. Rather, we want to hear about your experiences and routines in relation to your health. As part of our discussion, I will ask you about the treatment you receive from healthcare professionals, and how your family, friends and wider community may or may not help you.

If at any point during the interview you feel uncomfortable, please let me know and we can stop the interview.

1. I would like to start by asking you about yourself:
   1. Where were you born and where did you grow up?
   2. Did you go to school?
   3. Who do you live with?
   4. What do you do on a normal day?
2. Please can you tell me about any long-term medical conditions that you have been diagnosed with by a doctor or health facility:

(you can prompt for eg hypertension, diabetes, HIV, epilepsy, arthritis, chronic pain, lung disease/chronic cough including asthma, liver disease, blindness)

- 1. When were you diagnosed with each condition?
  2. What happened after you were diagnosed – did you receive medication? How about advice relating to your lifestyle?

1. Did your life change in any way after you were diagnosed with [conditions]?
   1. Did you have to adjust your routines e.g. work, household duties, food and drink consumption?
   2. Did your family do anything different after you were diagnosed?
   3. How about your friends and the wider community – did they change anything after you were diagnosed with [conditions]?
2. After you were diagnosed with [conditions], did the way you see yourself change at all?
   1. If so, how?
   2. Did your mood change?
   3. Did your reaction to [conditions] vary? If so, how?
3. Do you go to receive medical care for each of the conditions you have been diagnosed with?
4. If so,
   1. How far do you travel to these appointments?
   2. How do you get there?
   3. Does anyone help you travel?
   4. Do you see different doctors for each condition?
   5. How often are each of these appointments?
   6. How often do you miss appointments.
5. Do you take any medications for [conditions]?
   1. What are these medications?
   2. Where do you get them from?
   3. How do you pay for them?
6. Does anyone help you to take care of yourself?
   1. Family? If so, how?
   2. Friends? If so, how?
   3. Wider community? If so, how?
   4. Has any family member moved to live with you to help take care of you?
7. Do you help to take care of anyone else in your household who has a long-term condition?
8. Overall, can you summarise by telling me about any challenges you face living with multiple long-term conditions?
