# Supplementary material for: Experiences of multimorbidity in urban and rural Malawi: An interview study of burdens of treatment and lack of treatment
Source: PLOS Glob Public Health. 2022 Mar 24;2(3):e0000139. doi: 10.1371/journal.pgph.0000139 (PMC10021162; doi:10.1371/journal.pgph.0000139)
Supplement: Attachment — Submitted filename: Reviewer comments FINAL.docx [file pgph.0000139.s003.docx]

| **Reviewer’s comments** | **Our response** |
| --- | --- |
| Abstract: The abstract mentions “navigating health systems” twice. In the abstract conclusion, it would be better to put the utility of NPT and BOTT after the main findings, in line with how the study was structured and presented. | We have removed the duplication – thank you for spotting the error  We have made the adjustment as suggested and agree it flows better this way. |
| Introduction: The introduction is well written overall and presents key figures for context. In some places, however, the authors have used a number of references to support a single statement (see lines 88-89 as an example). It would be worthwhile retaining the 1-2 most relevant or recent references in this situation. | Thank you. We have reduced the number of supporting references by removing the original references 1, 8, 10, 14, 18 and 21 from the revised introduction, giving preference to the most relevant or recent references as suggested while still keeping sufficient references to demonstrate the breadth and weight of the evidence behind our statements. |
| In the opening paragraphs, Sub-Saharan Africa is mentioned however the authors quickly move to Malawi in the text that follows. It would be good to discuss the wider context before Malawi is brought into focus. Doing so, it is hoped, would provide a reasonable justification for conducting this work in Malawi. | We agree that the introduction would flow better this way and have re-ordered the introduction in line with the reviewers comments so that discussion of SSA issues come prior to mentioning Malawi specific details. |
| Available evidence on multimorbidity in Malawi seems to suggest a very low burden, can authors provide a brief commentary on this (single sentence) before moving on? | We agree that commentary is needed and have added a sentence and have added:  ‘These estimates are undoubtedly conservative, given high prevalence of HIV in these communities.’ |
| Methods: There needs to be more contextual and methodological detail around the process of recruitment and data collection, for the readers’ clarity. It is not clear for example, how and where participants were approached and recruited, where the data was collected and under what arrangements. Furthermore, authors should also provide some detail on the development of the interview guide. | We agree and thank the reviewer for pointing this out. We have added a substantial paragraph detailing the recruitment process:  ‘Potential participants were telephoned and asked if they would accept a visit from a researcher who would like to introduce a new study to them. If they agreed, a researcher visited the home of the potential participant and presented the study in vernacular language, using vernacular information sheets and encouraged participants to ask questions. A vernacular consent form was then read through with the participant and discussed. Written consent or thumb-print consent witnessed by a person of the participant’s choosing was given by all participants. ‘ |
| Results: The analysis is presented in sufficient detail elaborating all the identified themes. However, the authors frequently present a descriptive summary of their findings which is backed by an excerpt suggesting the same thing. For analytic depth, authors are recommended to keep this repetition to a minimum while also reducing the length of the texts used as excerpts. | We thank the reviewer for this comment and we have considered it very carefully within our team. However, in the same way clinical trials or epidemiological studies present key findings in the text, supported by tables or figures to illustrate and support the assertions being made, qualitative research requires that data, substantiating assertions underpinning reported findings are presented. Therefore we wish to maintain our current way of presenting our findings, which is in line with other papers reporting qualitative findings.  Nevertheless, we havereviewed all the included text carefully as requested and abbreviated wherever possible, avoiding the loss of meaning or key contextual data. |
| The paper suffered from wordiness in some sections; for example, and particularly, at Mobilising capacity 1.2. This section and others could do with a little tightening to reduce the wordiness. | Thank you we have read the manuscript carefully to try to sharpen the text and have shortened the specified section about Mobilising Capacity 1.2 as well as other sections of that table. |
| Ufa woyera is not 'fermented' but corn or maize floor made from de-husked corn/maize | We have made this change, thank you for pointing this error out. |
| The paper could be improved by a very precise statement of the goal(s) of the study, and then reporting on the methods and results for all goals set, and finally a reflection on their meaning and a compatible conclusion. | We have rephrased our aims/goals to make them clearer. In our introduction we now state:  “In this paper we aim to investigate: 1) how those living with multimorbidity in urban and rural Malawi describe their experiences of life with multimorbidity and particularly their experiences of burden of treatment; and 2) how useful NPT and BOTT are for structuring analytical accounts of these experiences.”  **In our methods we state that:**  We used semi-structured in-depth interviews to generate rich narratives of participants’ experiences of living with multimorbidity and burden of treatment which allowed us to better understand their experiences of burden of treatment. **(addressing aim one)**  And  To consider how useful NPT and BOTT are for understanding accounts of multimorbidity in Malawi, we first mapped themes onto a previously described burden of treatment framework using four NPT constructs (29, 30) namely: making sense of multimorbidity (coherence); interacting with others (cognitive participation); enacting management strategies (collective action); and reflecting on management (reflexive monitoring). We assessed whether each theme was able to ‘fit’ one (or more) of the four domains or whether it fell outside the framework. Our analysis is presented using these four NPT domains. We also drew on the extended conceptual framework provided by BoTT to understand what influenced capacity to cope with any given level of treatment burden and to summarise the findings **(addressing aim two).**  **In the first part of our discussion we now say:**  These findings illuminate the multiple and intersecting aspects of burden of treatment which people living with multimorbidity in urban/rural Malawi experience in their daily lives. We have shown that the key concepts of burden of treatment: gaining an understanding of life with multimorbidity (coherence); the engagement work of seeking family and community support (cognitive participation); enacting self-management advice (collective action); and appraising treatments (reflexive monitoring) identified through NPT and BOTT are described by people with multimorbidity in SSA. However, we have also shown that dealing with the burden of *lack* of treatments/services is a key issue.  Future work should therefore examine the processes through which this burden manifests in greater detail.  We have shown a similar interplay between treatment burden and capacity issues in SSA to that seen in HICs and that these issues can be usefully assessed and unpicked using both NPT and BoTT approaches, which highlight the social processes through which capacities and resources interact to produce greater or lesser experiences of burden. The work illustrates how poverty and inadequate healthcare and information provision in SSA constrained capacity to deal with treatment burden while supportive social and community networks were important enabling features.  **Conclusion**  Our study contributes to the emerging literature on experiences of multimorbidity in SSA and LMIC countries more broadly and demonstrates the utility of NPT and BOTT for aiding conceptualisation of treatment burden issues in LMICs. However, our findings highlight that ‘lack’ of access to treatments or services is an important additional dimension of the concept of burden of treatment which merits further investigation and should be integrated into future measures of treatment burden in SSA and likely other LMICs. Our work has shown that greater access to health information and education would lessen treatment burden as would better resourced healthcare systems that provide more continuity. Poverty also constrained capacity to deal with treatment burden suggesting the need for improved standards of living as a key issue. Nonetheless, supportive social and community networks were important enabling features and policies to support these networks would be important. |
| From the Abstract and Introduction, it’s clear to me that investigating the experiences of Malawi people with multimorbidity is an important study goal. However, it’s not clear to me whether or not “investigating their treatment burden of multimorbidity” is a study goal on itself. There is a clear focus though on the multimorbidity ‘burden’ throughout the paper. | Thank you for this comment we have made this clearer in the abstract which now reads:  We therefore explore: 1) experiences of men and women living with multimorbidity in urban and rural Malawi including their experiences of burden of treatment and 2) examine the utility of Normalization Process Theory (NPT) and Burden of Treatment Theory (BOTT) for structuring analytical accounts of these experiences.  And as outlined in response above have made this clearer in introduction and methods. |
| The Introduction states that “investigating the usefulness of NPT and BoTT for structuring the analytic accounts of these patients’ experiences” is another goal of this study. I find this goal hard to grasp. Unfortunately, the following parts of the paper do not succeed in explaining (to me) the use of this study goal (what does this research question mean and why is it a relevant question?), the way how exactly the researchers investigated this question, neither in what it resulted. If studying “the burden of multimorbidity” is aimed, I think it makes sense to use these instruments that have already shown their value, although in different settings. It seems to me that the researchers had good reasons to study “the multimorbidity burden” in Malawi, aided by NPT and BoTT, to assess similarities and differences in these patients’ experienced “burdens” as compared to patients with multimorbidity in non-SSA countries. | Thank you for this comment. NPT and BOTT have been developed through analysis of the reported experiences of people living with a range of chronic conditions and multimorbidity in high income countries, sometimes in countries like the UK where free universal access to healthcare is the norm. BOTT has emphasised how patient and caregiver workload and capacity are shaped by interactions between system level mechanisms that shape opportunities to obtain and utilize care and micro level mechanisms that shape individual agency, distributed social relations, and social capital that provide resources on which they can draw for support. An important implication of these models is that disparities between the self care work that is delegated to patients and caregivers, and their capacity to obtain and mobilise resources to do this work, may lead to structurally induced non adherence to self care regimens. Thus, these theories offer possible explanations for observed challenges at the system level and with self care at the micro level. It is likely, as we have shown in this paper, that while many of the basic mechanisms at play that influence burden of treatment and capacity may be generic (and apply across the HIC and LMIC contexts) that there may also be key differences in systems, culture and relationships in LMICs that mean these models may need to be refined or adapted as we have shown in this paper.  We know that these theories have shown their value in HICs, but we believe it was important for researchers to empirically determine that they have applicability in LMICs and to explore whether any refinements would be necessary to make them culturally appropriate for use in the LMIC context.  Our work has indeed shown that these theories can be usefully utilized in SSA/LMICs but has also shown that the theories need refined, as we have outlined in this paper.  To make this clearer we have slightly rewritten the introduction as below:  “NPT and BoTT seem to have the potential to support a generalizable understanding of multimorbidity in LMICs such as Malawi, but were developed in high-income settings and may not be either appropriate or relevant to LMICs such as Malawi, in that observed challenges for patients and their support community may differ at the system or individual level and the same may be true for factors that influence capacity. In this paper we aim to investigate: 1) how those living with multimorbidity in urban and rural Malawi describe their experiences of life with multimorbidity and particularly their experiences of burden of treatment; and 2) how useful NPT and BOTT are for structuring analytical accounts of these experiences, and whether these theoretical frameworks need any refinements or adaptation to increase their utility in a LMIC context. “  Similarly in the methods we have added the following:  “We also drew on the extended conceptual framework provided by BoTT to understand what influenced capacity to cope with any given level of treatment burden, to summarise the findings, and to identify any potential gaps in the theory” |
| The discussion of the paper needs some improvement / rewriting. Generally, this section of the paper starts with a summary (what again was the study goal, which are the results we found). The paragraph that is meant to compare the findings to existing literature, reveals to me an important novelty, a strength of the current study, namely the assessed importance of social networks and resources affecting the multimorbidity burden as, now for the first time, assessed in a SSA country, by applying the theories of NPT and BoTT, and revealing a new awareness, namely the burden of ‘lack of treatment’. The authors may consider writing an ‘implications for practice (and science)’ paragraph and label it accordingly. | Thank you for this comment. We have rewritten the first section of the discussion to address this comment and have also sharpened up the final conclusion as requested and outlined below.  The new first part of the introduction now reads as follows:  “These findings illuminate the multiple and intersecting aspects of burden of treatment which people living with multimorbidity in urban/rural Malawi experience in their daily lives.  We have shown that the key concepts of burden of treatment: gaining an understanding of life with multimorbidity (coherence); the engagement work of seeking family and community support (cognitive participation); enacting self-management advice (collective action); and appraising treatments (reflexive monitoring) identified through NPT and BOTT are described by people with multimorbidity in SSA. However, we have also shown that dealing with the burden of *lack* of treatments/services is a key issue. Future work should therefore examine the processes through which this burden manifests in greater detail.  We have shown a similar interplay between treatment burden and capacity issues in SSA to that seen in HICs and that these issues can be usefully assessed and unpicked using both NPT and BoTT approaches, which highlight the social processes through which capacities and resources interact to produce greater or lesser experiences of burden. Our work illustrates how poverty and inadequate healthcare and information provision in SSA constrained capacity to deal with treatment burden while supportive social and community networks were important enabling features.”  We have not added a specific “implications for practice and science paragraph” as that does not seem to fit with the journal style. However, we agree this would be useful and we have therefore reworked the conclusion, so it highlights the implications for practice and science. Please see new conclusion outlined in response to your specific query on this point. |
| The strengths and limitations section is not highly reflexive and inaccurately written (it states that 4 conditions were included – the methods state only 3). Some items discussed in lines 214-224 could be dealt with here. | Thank you for spotting our error – we have fixed this and now state three conditions. In relation to the reflexivity query, we prefer to follow the SRQR guidelines, which position the reflexivity section in the methods – we elaborate on this again below. |
| When reverting to the exact research question(s), also the conclusion paragraph could be somewhat more concise and crisp. And it could correspond somewhat better with the conclusion as stated in the abstract. | Thank you for this. We have rewritten the conclusion as suggested.  It now reads as follows:  “Our study contributes to the emerging literature on experiences of multimorbidity in SSA and LMIC countries more broadly and demonstrates the utility of NPT and BOTT for aiding conceptualisation of treatment burden issues in LMICs. However, our findings highlight that ‘lack’ of access to treatments or services is an important additional dimension of the concept of burden of treatment which merits further investigation and should be integrated into future measures of treatment burden in SSA and likely other LMICs. Our work has shown that greater access to health information and education would lessen treatment burden as would better resourced healthcare systems that provide more continuity. Poverty also constrained capacity to deal with treatment burden suggesting the need for improved standards of living as a key issue. Nonetheless, supportive social and community networks were important enabling features and policies to support these networks would be important. |
| In the results section, the citations do not always clearly illustrate that what is stated by the authors. For example: line 538 and the citation following. Line 551 and the following citation is another example where the citation gives quite general statements and does not provide tangible ‘solutions’. | The citations we have used in these instances reference the paper in which BoTT concepts are set out. We have looked carefully through the manuscript to see where we have used references to refer to concepts in the original papers describing BoTT concepts and explained their use. |
| The paper might benefit from comments from the authors on non-verbal communication (if studied / recorded). | We thank you for this suggestion, but we did not study/record non-verbal communication in this study. |
| Reflection on cultural aspects (in the discussion) might be helpful for western readers of this study. For example, important factors when interviewees comment on organisations and persons with authority such as doctors, governments. Or on the communication between interviewer and interviewee, e.g. if inconsistencies would be noticed (money might enable patients but doesn’t immediately solve the issue of unavailability). | We thank you for this comment and believe that our revised discussion draws out a key context-specific finding: the burden of lack of treatment.  We have added a limitation relating to power imbalances between community members and authority figures/bodies such as medical professionals: "Finally, while many interviewees gave critical accounts of the care they receive, not all did; it is possible that some feared repercussions from healthcare professionals and other authorities or that respect for those in professional positions tempered criticism of difficult experience.”  Thank you for this observation about communication between interviewers/interviewees. We believe that the communication achieved was of a high standard, given that the interviewers are native speakers who live or have lived in the communities in which the work was carried out. However, we have tried to make the discussion of medication availability clearer by noting that the lack we discuss related to ‘obtaining medicines **from public hospitals**.’ Those who have money are able to circumvent this lack by purchasing medicines directly form pharmacists, as described on lines 708-717. |
| Line 222-223: comments such as these should be more appropriately placed elsewhere (not in the Methods section). | We were guided by [Standards for Reporting Qualitative Research Guidelines](https://edisciplinas.usp.br/pluginfile.php/4983308/mod_resource/content/1/SRQR_Standards%20for%20Reporting%20Qualitative%20Research_OBrien_2014.pdf) which suggests that techniques to enhance trustworthiness of analysis, which include reflexivity, should be presented in the methods. These standards are recognised by the journal as one of two acceptable reporting standards for qualitative research. We have, then, left discussion of reflexivity in the methods section but return to it, as discussed above, in the limitations sections where we discuss potential explanations for lack of criticism of the health system. |
| Line 242: I see it as a respectful and appropriate step to mention that community leaders were asked permission (Ethics paragraph). | Thank you, we appreciate this. |
| Line 528-532: reading this quotation, labelled as ‘choosing care providers’ and concerning ‘stigma’, to me the question arises whether ‘low trust in, or concerns on doctor’s professionality’ (disclosing confidential patient information to others) could be seen as a (recurrent) theme, maybe also in other interviews? I wonder what the author’s view is on this issue and how it impacts (or doesn’t impact) the burden of multimorbidity. | Thank you for this interesting reflection. However, this extract is not about trust in doctors or health professionals, but about being *seen* at a local clinic by other community members and the consequences this has for the person living with multimorbidity. We have made some adjustments to the text here to make this clearer ‘Being seen attending a clinic was a focal concern’ – see ines 644-649 |
| The abstract contains a duplication in the results. It could focus more on the new findings in Malawi, compared to already known aspects defining the multimorbidity burden from previous studies (in western countries). | We have removed the duplication, thank you. To address the point about focusing on new findings, rather than rewrite the results section of the abstract we have re-ordered the conclusion bringing out the new findings first. |
| **Journal Comments** | **Response** |
| 1. Please provide additional details regarding participant consent. In the ethics statement in the Methods and online submission information, please ensure that you have specified what type you obtained (for instance, written or verbal, and if verbal, how it was documented and witnessed). If your study included minors, state whether you obtained consent from parents or guardians. | We have added a clear statement that written consent was obtained on line 183, specifically, we have written:  “Potential participants were telephoned and asked if they would accept a visit from a researcher who would like to introduce a new study to them. If they agreed, a researcher visited the home of the potential participant and presented the study in in participants’ everyday language vernacular language (Chichewa or Chitumbuka), using information sheets (also in the vernacular) and encouraged participants to ask questions. A vernacular consent form was then read through with the participant and discussed. Written consent or thumb-print consent witnessed by a person of the participant’s choosing was given by all participants.” |
| 2. In the online submission form, you indicated that "All anonymised data relevant to the study are available from the authors on reasonable request."  All PLOS journals now require all data underlying the findings described in their manuscript to be freely available to other researchers, either 1. In a public repository, 2. Within the manuscript itself, or 3. Uploaded as supplementary information.  This policy applies to all data except where public deposition would breach compliance with the protocol approved by your research ethics board. If your data cannot be made publicly available for ethical or legal reasons (e.g., public availability would compromise patient privacy), please explain your reasons by return email and your exemption request will be escalated to the editor for approval. Your exemption request will be handled independently and will not hold up the peer review process, but will need to be resolved should your manuscript be accepted for publication. One of the Editorial team will then be in touch if there are any issues. | The information sheet and consent form approved by the RECs and used in the study do not give us license to make the study data publicly available. However, the statement we indicated in the submission form stands: we can share anonymized data with bona fide researchers on reasonable request. |
| 3. State what role the funders took in the study. If the funders had no role in your study, please state: “The funders had no role in study design, data collection and analysis, decision to publish, or preparation of the manuscript.” | This phrasing is now used on lines 894-898.  We write:  This work was funded by the MRC Grant MR/T037849/1 and by the University of Glasgow’s GCRF Small Grants Fund, an initiative supported by an allocation of Global Challenges Research Fund from the Scottish Funding Council under agreement SFC/AN/10/2018. The funders had no role in study design, data collection and analysis, decision to publish, or preparation of the manuscript. |
